# Maternal obesity impairs skeletal development in adult offspring

**DOI:** 10.1530/JOE-18-0244

**Published:** 2018-07-24

**Authors:** Jin-Ran Chen, Oxana P Lazarenko, Haijun Zhao, Alexander W Alund, Kartik Shankar

**Affiliations:** 1 Arkansas Children’s Nutrition CenterLittle Rock, Arkansas, USA; 2 Department of PediatricsUniversity of Arkansas for Medical Sciences, Little Rock, Arkansas, USA; 3 Interdisciplinary Biomedical Sciences University of Arkansas for Medical Sciences, Little Rock, Arkansas, USA

**Keywords:** osteoblast, senescence, obesity, high-fat diet, epigenetic

## Abstract

Intrauterine or early postnatal high-fat diet (HFD) has substantial influences on adult offspring health; however, studies of HFD-induced maternal obesity on regulation of adult offspring bone formation are sparse. Here, we investigated the effects of HFD-induced maternal obesity on both fetal and adult offspring skeletal development. We found that HFD-induced maternal obesity significantly decreased fetal skeletal development, but enhanced fetal osteoblastic cell senescence signaling and significantly increased the expression of inflammatory factors of the senescence-associated secretory phenotype (SASP) in osteo-progenitors. It was found that p300/CBP activation led to H3K27 acetylation to increase the expression of senescence-related genes and PPARγ in embryonic mouse osteogenic calvarial cells from HFD obese dams. These results were recapitulated in human umbilical cord mesenchymal stem cells (UC MSCs) isolated from offspring of pregnant obese and lean mothers following delivery. Regardless of postnatal HFD challenge, adult offspring from HFD obese dams showed significantly suppressed bone formation. Such early involution of bone formation of adult offspring from HFD obese dams may at least in part due to histone acetylation, i.e., epigenetic regulation of genes involved in cell senescence signaling in pre-osteoblasts from prenatal development. These findings indicate fetal pre-osteoblastic cell senescence signaling is epigenetically regulated by maternal obesity to repress bone formation in adult offspring in rodents and suggest that at least some of these effects may also manifest in humans.

## Introduction

Expression of certain genes in the genome can be regulated without altering the base sequence of DNA: e.g., through chromatin remodeling, DNA methylation, histone modifications (acetylation) and the expression of non-coding RNA (Vrtačnik *et al*. 2014). These mechanisms of epigenetics are important in regulating differentiation of different types of cells during both prenatal and postnatal development. Maternal nutrition appears to influence epigenetic alterations in the offspring and to program gene expression in key metabolic pathways, such as fatty acids and glucose metabolism (Levin & Govek 1998, King 2006, Shankar *et al*. 2008, Geraghty *et al*. 2016). Emerging evidence suggests that maternal obesity caused by excessive consumption of a high-calorie, high-fat diet (HFD) has a profound influence on the health of cardiovascular and skeletal system of the offspring during fetal development and infancy, as well as later on in childhood and into adulthood (Barker 2007, Samuelsson *et al*. 2008, McCurdy *et al*. 2009). The relevance of epigenetic control in bone-related gene expression, bone development and organ homeostasis, as well as in the onset and progression of musculoskeletal diseases, has also been increasingly shown (Lian 2015). We have recently reported the effects of maternal HFD on fetal osteogenic cell differentiation behaviors (Chen *et al*. 2012, 2016); however, the influence of HFD-induced maternal obesity on epigenetic regulation of offspring adult bone development has not been proven.

Bone mineralization within the skeletal envelope is impacted by variety of factors such as local paracrine molecules and hormones traveling through the bloodstream (Riggs *et al*. 2002), dietary factors and lifestyle, such as the amount of physical activity. Intrauterine programming of bone development leading to increased risk of osteoporosis in adulthood has been recently suggested (Dennison *et al*. 2013). Although there are no convincing data on determining the effect of obesity on early postnatal life bone quantity (Streeter *et al*. 2013, Kâ *et al*. 2013), feeding a HFD rich in saturated fat and cholesterol to experimental rodents has consistently been shown to inhibit bone formation (Parhami *et al*. 2001, Rosen & Bouxsein 2006, Zhao *et al*. 2008, Liang *et al*. 2009, Chen *et al*. 2010, 2013). Previous animal data indicated that inhibition of perinatal skeletal formation by HFD consumption during gestation (Liang *et al*. 2009, Chen *et al*. 2012) might be associated with DNA hypermethylation, suggesting intrauterine fetal skeletal developmental programming (Chen *et al*. 2012). We have recently provided evidence showing maternal HFD-induced obesity accelerates senescence signaling and therefore leads to altered glucose metabolism and mitochondrial function in osteo-progenitors derived from rats and humans (Chen *et al*. 2016), while these remain unrevealed in bone in postnatal adult offspring.

Cellular senescence is a general process, and it is thought to occur in most cells of organisms during aging and most notably within tumors (Campisi & d’Adda di Fagagna 2007, Collado *et al*. 2007). However, it was recently identified during mammalian embryonic development (Muñoz-Espín *et al*. 2013, Storer *et al*. 2013), leading to our interests in investigating links between gestational obesity and fetal cell senescence programming. In non-cancer cells, the (patho) physiologic role of cell senescence signaling is currently unknown. Although many senescence mediators have been characterized in rodent cells, induction of tumor suppressor genes p53, p21 and p16 are critical to the induction of senescence. Senescent cells increase the expression and secretion of numerous cytokines, chemokines, matrix metalloproteinases and other proteins that can alter local tissue environments. This feature was termed the senescence-associated secretory phenotype (SASP) (Coppé *et al*. 2008); this may be tracked by senescence-associated β-galactosidase (SAβG) activity, the most widely used marker to detect senescence both *in vitro* and *in vivo* (Dimri *et al*. 1995, Collado & Serrano 2006).

Relevant to the role of p300/CBP histone acetyltransferases (HATs), hyperacetylation of histone N-terminal tail lysines correlates strongly with active gene transcription (Bose *et al*. 2017). p300/CBP are together chiefly responsible for the global acetylation of histone H3 residues K18 and K27 and contribute to other locus-specific histone acetylation events. As in other tissues, intrauterine fetal skeletal programming is complex and may undergo postnatal reprogramming; however, histone modification by acetylation has been shown to be important for long-term regulation of cellular function (Kruhlak *et al*. 2001). This regulation occurs by inducing an open chromatin structure that correlates with gene activation. H3K-associated fetal tissue programming may affect several generations (Rando & Chang 2009) making it a difficult phenomenon to target for disease intervention. It remains unclear if an increase in H3K27 acetylation leads to epigenetic overexpression of target genes in embryonic osteoblastic cells and in bone of adult offspring. We hypothesize that HFD-induced maternal obesity increases H3K27 acetylation in osteogenic cells to change osteoblastic cell differentiation and activity.

## Materials and methods

### Animals and diets

Female 4-week-old C57BL6/J mice were purchased from Jackson Laboratory (https://www.jax.org/). Subsequently, these mice were divided into two groups: one group of mice received control semi-purified AIN-93G control diet (17% fat diet corn oil) and the other group of mice received a high-fat diet (by calories, 25% protein, 45% fat, and 30% carbohydrates). After 12 weeks of diet intervention (pre-pregnancy), mice were time-impregnated (*n* = 12 per group) by control male mice; this HFD-induced maternal obesity mouse model was similar to what we described previously in rats (Chen *et al*. 2012). Pregnant mice were individually housed in an Association for Assessment and Accreditation of Laboratory Animal Care-approved animal facility at the Arkansas Children’s Research Institute (Little Rock, AR) with constant humidity, lights on from 06:00 to 18:00 h, at 22°C. All animal procedures were approved by the Institutional Animal Care and Use Committee at University of Arkansas for Medical Sciences (UAMS, Little Rock, AR). One set of pregnant HFD and control diet mice were killed, and embryos were taken at gestational day 17.5 for analysis. The remainder of pregnant HFD and control diet mice were continued on diet regimens until pups were born. Male and female offspring from either HFD or control diet dams were weaned at 21 days of age onto either HFD or control diet, and diets were continued for 14 weeks.

### Pregnant lean and obese women, and isolation of human UC MSCs

Twenty-four pregnant women were recruited for this study: twelve obese (pre-pregnancy BMI ≥30 kg/m^2^) and twelve non-obese (pre-pregnancy BMI between 19 and 25 kg/m^2^). After obtaining written informed consent during the first trimester of pregnancy, placentas and umbilical cords (UCs) were collected at UAMS following delivery. The protocol was approved by the Institutional Review Board at the UAMS. As described previously (Saben *et al*. 2014), mesenchymal stem cells (MSCs) from the UC matrix were isolated from the UCs, pooled and expanded. UC MSCs were counted and plated in growth media in a single well of a six-well plate. These UC MSCs were used for experiments described in the ‘Results’ section, and we utilized a subset of cells randomly from three obese mothers and from three lean mothers for the analysis in the current report.

### Fetal and postnatal adult offspring tibial micro-computed tomography scan

We are not aware of any references of micro-computed tomography (μCT) for embryos; therefore, we used standard procedures for mouse tibia (see below), and bone volume was the only parameter analyzed. Adult offspring tibial μCT measurements of the trabecular and cortical compartments from the right tibial bone were evaluated using a Scanco μCT scanner (μCT-40; Scanco Medical AG, Bassersdorf, Switzerland) at 6 μm isotropic voxel size with X-ray source power of 55 kV and 145 μA and integration time of 300 ms. The gray-scale images were processed by using a low-pass Gaussian filter (*σ* = 0.8, support = 1) to remove noise, and a fixed threshold of 220 was used to extract the mineralized bone from the soft tissue and marrow phase. Cancellous bone was separated from the cortical regions by semi-automatically drawn contours. A total of 120 slices starting from about 0.1 mm distal to growth plate, constituting 0.70 mm of length, was evaluated for trabecular bone structure based on description by Bouxsein* et al*. (2010), and by using software provided by Scanco, as described in detail previously (Cao *et al*. 2009, Chen *et al*. 2009).

### Fetal Alizarin red/alcian blue staining and isolation of embryonic calvarial cells

The gold standard Alizarin red/alcian blue staining for observation of fetal skeletal development and mineralization was used according to a method published previously (Chen *et al*. 2012). Pregnant female mice were killed on day 17 postcoitum (with 0 considered the day of the positive vaginal smear), and fetuses were collected. For the isolation of fetal calvarial cells, collagenase digestion of calvarial tissue was performed and cells from second and third digestion were collected and pooled. The detailed procedure for isolation of neonatal mouse and rat calvarial osteoblastic cells as previously published (Chen *et al*. 2012).

### Cell cultures and real-time reverse transcription-polymerase chain reaction (real-time PCR)

Isolated fetal/embryonic mouse osteogenic calvarial cells (EOCCs) or human UC MSCs were cultured in α-MEM (# 41061-029, Invitrogen) supplemented with 10% fetal bovine serum (FBS) (Hyclone, Logan, UT, USA), penicillin (100 units/mL), streptomycin (100 µg/mL) and glutamine (4 mM). α-MEM supplemented with 10% FBS, 1 mM ascorbyl-2-phosphate (Sigma-Aldrich) and 4 mM l-glutamine was used as osteoblast (OB) differentiation medium, while α-MEM supplemented with 10% FBS was used as control medium. Cell RNA from isolated *in vitro*-cultured cells and *in vivo* adult offspring bone tissue RNA were extracted using TRI Reagent (MRC Inc., Cincinnati, OH, USA) according to the manufacturer’s recommendation followed by DNase digestion and column cleanup using QIAGEN mini columns (Chen* et al*. 2016). Reverse transcription was carried out using an iScript cDNA synthesis kit from Bio-Rad. All primers for real-time PCR analysis used in this report were designed using Primer Express software 2.0.0 (Applied Biosystems).

### Mouse and human cell SAβG activity assay, histology and immunostaining

Protein senescence-associated β-galactosidase (SAβG) activity assay was performed according to a method described previously (Thornburg *et al*. 2010). SAβG staining was performed on cultured cells either from HFD obese mice or control mice and on human UC MSCs from either obese or lean mothers using four-well chamber slides. The SAβG staining procedure was described previously (Chen *et al*. 2016). Standard antibody counterstaining for p53 and p21 (Cell Signaling) was performed after SAβG staining.

### Western blot, inflammation antibody array and chromatin immunoprecipitation (ChIP)

Standard Western blots were performed using total protein isolated from cultured cells and bone tissue after aspiration of bone marrow. The following antibodies were used for Western blots: H3k27ac (#07-360, Millipore), p300/CBP (#P2859, Sigma), p300 (#05-257, Sigma), CBP (#SAB4500455, Sigma), β-actin (#A1978, Sigma), PPARγ (#ab191407, Abcam), and osteocalcin (#AB10911, Millipore). SuperSignal West Pico chemiluminescent substrate (Pierce) was used for developing blots. Mouse (#AAM-INF-1-8, RayBiotech, Inc) inflammation antibody array was performed according to the protocol provided by manufacturer. Procedure for standard ChIP assay using H3K27ac antibody (ChIP grade from Cell Signaling) was described previously (Chen *et al*. 2010), and information for all primers used for ChIP assay were listed in Supplementary Table 1 (see section on supplementary data given at the end of this article).

### Measurements of bone formation markers and glucose and insulin levels in bone marrow plasma

Bone marrow plasma was prepared at the time of tissue harvest. Bone marrow was flushed out from the femur using 300 µL of PBS, vortexed and spun (1700 ***g***) for collecting supernatant as bone marrow plasma. The bone formation marker, bone-specific alkaline phophatase (ALP) was measured by Rat-MIDTM ALP ELISA (Nordic Biosciences Diagnostic, Herlev, Denmark). The bone marrow plasma total osteocalcin (Gla-OC) and undercarboxylated osteocalcin (Glu-OC) levels were measured by an ELISA-based kit from TAKARA (TAKARA Bio Inc.). The bone marrow plasma glucose and insulin levels were measured by Rat-LapsTM ELISA from Nordic Biosciences Diagnostic (Herlev, Denmark).

### Statistical analyses

Data were expressed as means ± standard error. One-way and two-way ANOVA followed by Student–Newman–Keuls *post hoc* analysis was used to compare the treatment groups. Values were considered statistically significant at *P* < 0.05.

## Results

### HFD-induced maternal obesity impairs embryonic skeletal development and cell senescence signaling in mice

**Figure 1:**
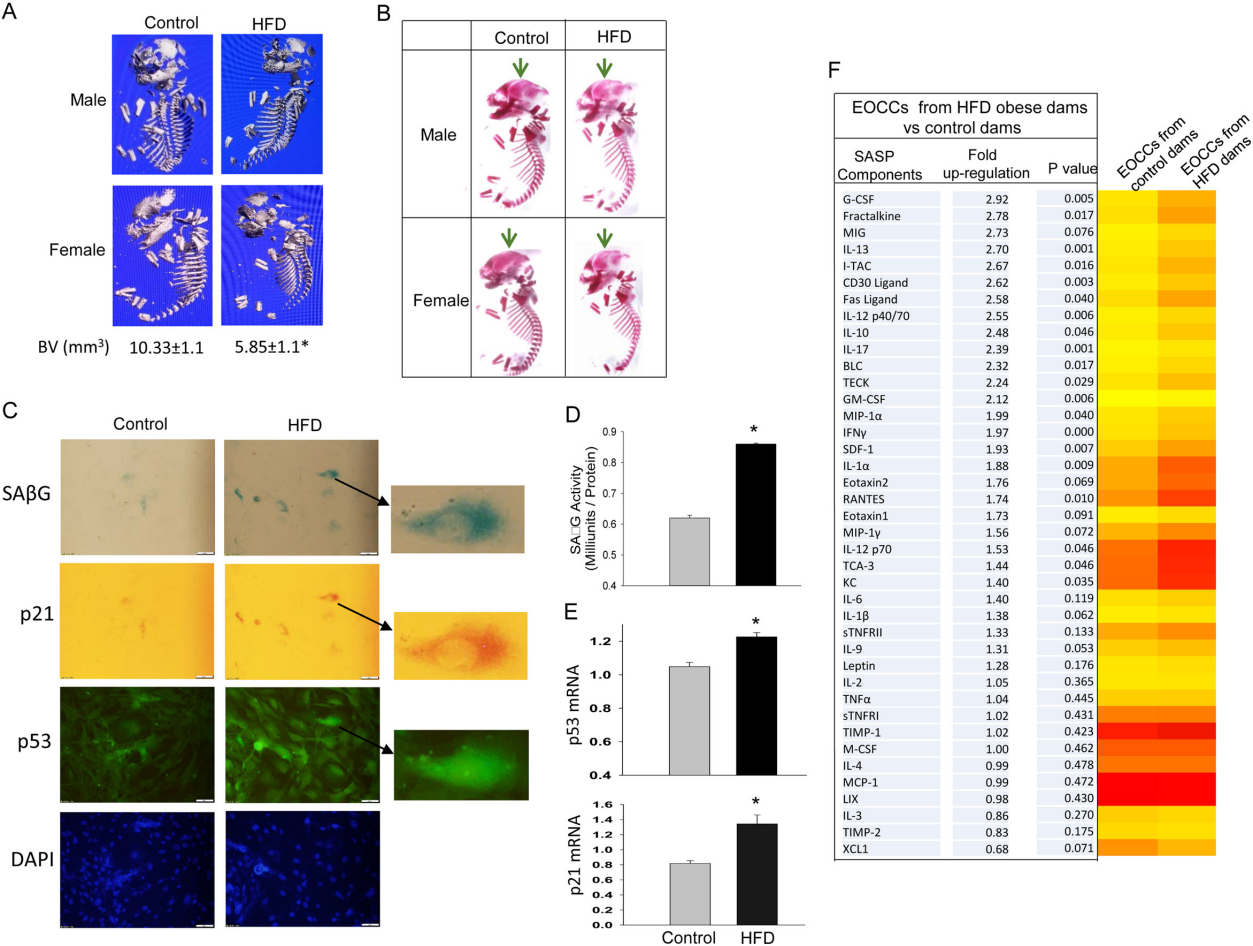
Delayed skeletal development and increased senescence in osteo-progenitors in fetuses from HFD (high-fat diet) mouse dams. (A) µCT for male and female embryos (whole fetus scan) either from six HFD-obese mouse dams (three male and three female) or six control diet mouse dams (three male and three female). The numbers under pictures are bone volume (BV) from fetus µCT. (B) Indicating impaired skeletal ossification in fetuses from HFD mouse dams. Alizarin red/alcian blue staining of GED 17.5 embryos either from control diet dams or HFD dams. Bone calcification stained red, and green arrows indicate differences of skeletal ossification in the head between embryos of control and HFD dams. (C) Isolated embryonic mouse osteogenic calvarial cells (EOCCs) either from HFD or control diet dams were cultured in chamber slides, triple-staining of senescence-associated β-galactosidase (SAβG) blue staining, and p21 (red) and p53 (green) antibody immune-staining were performed. (D) SAβG activity measurement in proteins from EOCCs either from HFD or control diet dams. (E) p53 and p21 mRNA expression in EOCCs. (F) Increased SASP in EOCCs from HFD obese dams. Proteins isolated from EOCCs either from control or HFD obese dams were analyzed by antibody arrays. Shown is the heat map analysis for comparison of all factors in EOCCs from control and HFD obese dams. *P* < 0.05 means factors are significantly increased in EOCCs from HFD obese dams compared to those from control diet dams. *P < 0.05 by *t*-test vs control.

A maternal obese mouse model was developed by feeding female C57BL6/J mice with HFD for 12 weeks pre-pregnancy and during pregnancy (Supplementary Fig. 1, experimental design). At gestational day 17 (E17.5), embryos were taken, and fetal calvarial forming cells were isolated for experimental analysis. Dam body weight gains during the HFD period until offspring began weaning were presented (Supplementary Fig. 1). Fetuses from both HFD-obese and control diet dams were first μCT scanned. A total of 12 fetuses were analyzed: six fetuses (three males and three females) from six different HFD obese dams and six fetuses (three males and three females) from six different control diet dams. While fetal body weights were not found to be significantly different between HFD obese and control diet dams, μCT showed significantly reduced total bone volume (BV) in embryos from HFD-obese dams compared to those from control diet dams (Fig. 1A). There were no differences in BV between male and female fetuses within either diet (data not shown). After μCT scan, we performed Alizarin Red Staining on whole fetal skeleton for osteogenesis or bone mineralization. We observed less bone mineralization in both male and female embryos from HFD-obese dams compared to those male and female embryos from control diet dams (Fig. 1B).

Fetal calvarial osteo-progenitor cells were isolated by using collagenase type 1 digestion of very thin calvarias (one time digestion). These cells are embryonic osteo-progenitors because under appropriate stimulations, these cells can differentiate either toward adipocyte-like cells or osteoblastic cells (Chen *et al*. 2012). The embryonic calvarial osteo-progenitor cells from either HFD obese dams or control diet dams were cultured in chamber slides. Using methods established previously (Chen *et al*. 2016), exploratory senescence-associated β-galactosidase (SAβG) staining and counterstaining using p21, p53 antibody immunostaining were performed. Increased senescence signals were found in embryonic calvarial osteo-progenitor cells from HFD obese dams (Fig. 1C). To confirm these data, we performed SAβG activity measurements in proteins isolated from embryonic calvarial osteo-progenitor cells. The SAβG activity was significantly higher in embryonic calvarial osteo-progenitor cells from HFD-obese dams compared to those from control diet dams (Fig. 1D). Increased SAβG activity was accompanied by increased gene expression of p53 and p21 (Fig. 1E), two well-known mediators associated with cellular senescence, in embryonic calvarial osteo-progenitor cells from HFD obese dams. Senescent cells increase the expression and secretion of numerous cytokines, chemokines, MMPs and other proteins that can alter local tissue environments; this feature has been termed the SASP. We evaluated the SASP for embryonic calvarial osteo-progenitor cells from HFD-induced obese dams compared to embryonic calvarial osteo-progenitor cells from control dams, using an inflammatory antibody array (Fig. 1F). Strikingly, half of these SASP molecules were significantly upregulated in embryonic calvarial osteo-progenitor cells from HFD-induced obese dams compared to embryonic calvarial osteo-progenitor cells from control dams (Fig. 1F). These data suggest that embryonic skeletal development in HFD obese dams is characterized by cellular senescence promotion and inflammatory milieus.

### Obesity-induced increases of senescence signaling in osteo-progenitors are associated with p300/CBP activation and H3K27 acetylation in both mice and humans

**Figure 2:**
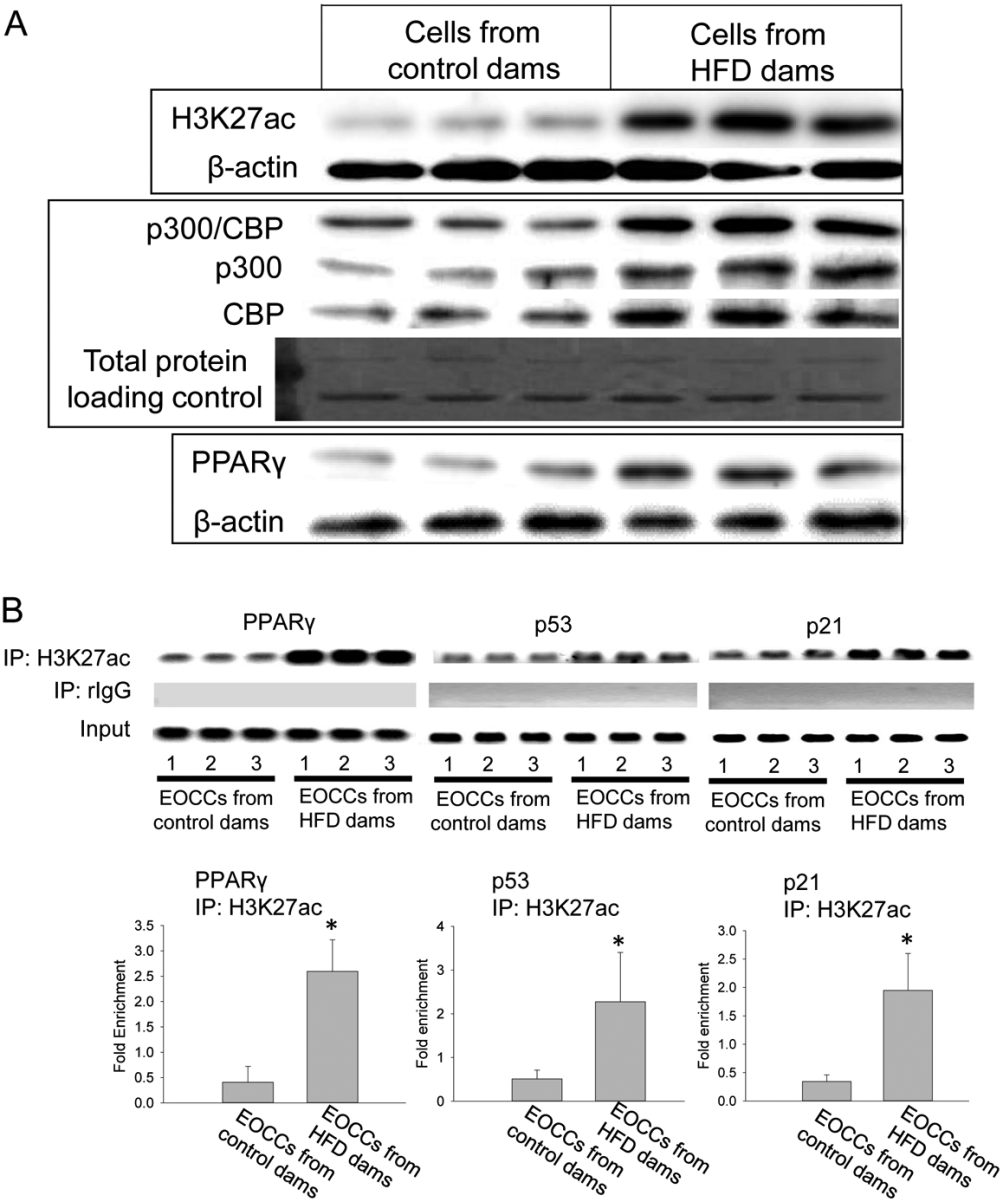
Identification of senescence-associated signaling pathway in EOCCs (embryonic mouse osteogenic calvarial cells) by maternal obesity during fetal skeletal development. (A) Proteins isolated from embryonic mouse osteogenic calvarial cells either from HFD or control diet dams (three per group); Western blots for H3K27ac, p300/CBP, p300, CBP and PPARγ. (B) ChIP of mouse PPARγ, p53 and p21 enhancer elements by specific anti H3K27ac antibody, and ChIP of enrichment of PPARγ, p53 and p21 after IP with H3K27ac antibody, fold enrichment relative to IgG. *P < 0.01 by *t*-test vs control.

Proteins were isolated from calvarial cells of a subset of six male fetuses from six different dams, three from control dams and three from HFD dams. Western blots showed remarkable increases in acetylation of lysine 27 on histone H3 (H3K27ac), and expression of p300/CBP proteins (Fig. 2A). It has been shown that modification of histone tails plays an important role on regulating gene transcription and that hyperacetylation of histone N-terminal tail lysines (H3K27 as an example) correlates strongly with activation of hundreds of transcription factors including nuclear receptors such as PPARγ. We found that PPARγ expression was increased in embryonic calvarial osteo-progenitor cells from HFD-induced obese dams (Fig. 2A). To determine whether HFD maternal obesity enhances binding of H3K27ac to PPARγ and cellular senescence-associated target genes (p53 and p21), H3K27ac chromatin immunoprecipitation (ChIP) assays were carried out (Fig. 2B). We used antibody against H3K27ac and subsequent PCR amplification. We found that there were pronounced increases in the bindings of H3K27ac to the PPARγ gene and to p53 and p21 in embryonic calvarial osteo-progenitor cells from HFD obese dams compared to those cells from control diet dams (Fig. 2B).

**Figure 3:**
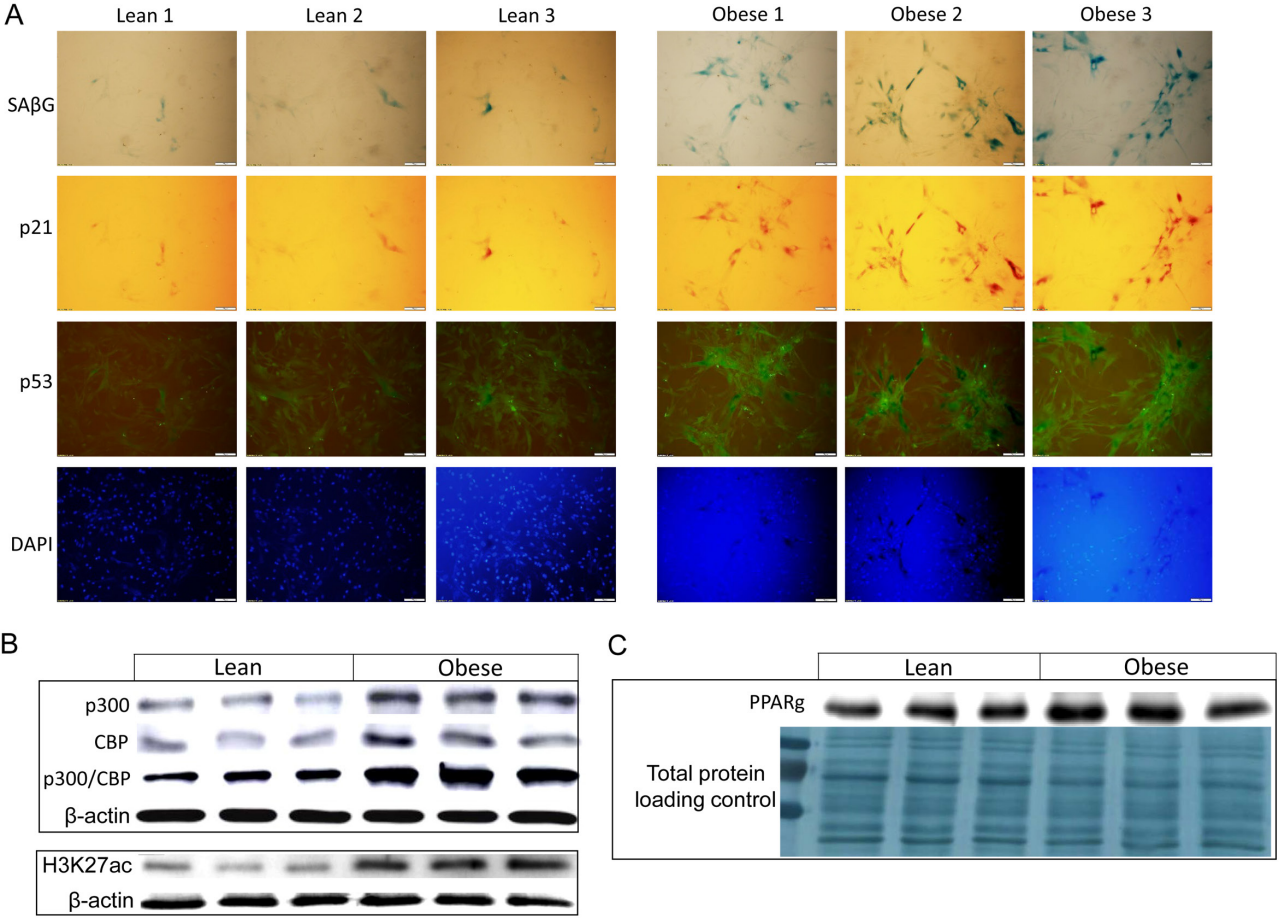
Increased senescence signaling in mesenchymal stem cells (MSCs) from umbilical cords (UC) of obese mothers. (A) Human UC MSCs either from three lean mothers or three obese mothers were cultured in chamber slides, triple-staining of senescence-associated β-galactosidase (SAβG) blue staining, and p21 (red) and p53 (green) antibody immune-staining were performed. (B) Proteins isolated from human UC MSCs either three lean mothers or three obese mothers, Western blots for p300, CBP, p300/CBP and H3K27ac, and (C) for PPARγ, Ponceau S staining on original membrane is showing for protein loading controls.

**Figure 4:**
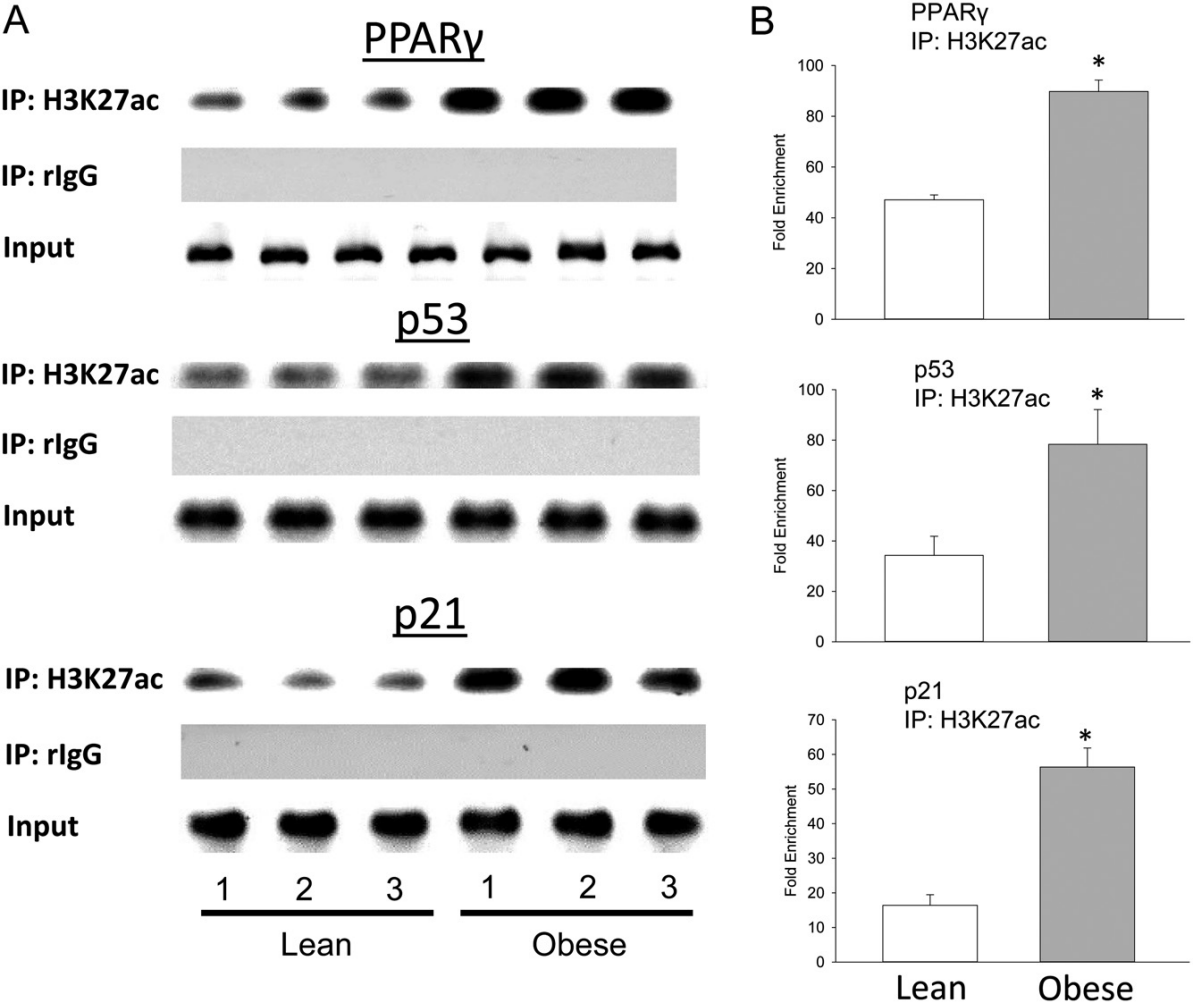
Increased association between H3K27ac and senescence molecules in human UC MSCs from obese mothers. (A) ChIP of human PPARγ, p53 and p21 enhancer elements by specific anti H3K27ac antibody, and (B) ChIP of enrichment of human PPARγ, p53 and p21 after IP with H3K27ac antibody, fold enrichment relative to IgG. *P < 0.01 by *t*-test vs lean.

Given the activations of H3K27 ac, p300/CBP, PPARγ and senescence signal changes in embryonic calvarial osteo-progenitor cells from HFD-induced obese mouse dams, we next considered if such events may occur in humans. Considering that embryonic calvarial osteo-progenitor cells are not feasible to study in humans, we studied senescence and p300/CBP/H3K27ac-associated epigenetic signals in isolated MSCs from human UCs. Experiments were conducted in human MSCs from a subset of obese (pre-pregnancy BMI ≥30 kg/m^2^) and control lean mothers (pre-pregnancy BMI between 19 and 25 kg/m^2^), random three per each group, following delivery. Information on the study subjects, the procedure and characterization of UC cells as MSCs have previously been described (Saben* et al*. 2014). UC MSCs were cultured in chamber slides. Using methods and similar approaches described earlier in cells from mice, exploratory SAβG staining and counterstaining using p21, p53 antibody immunostainings were performed. Clearly, when compared to non-obese mothers, (Fig. 3A) senescence protein markers in UC MSCs from obese mothers were substantially higher. Moreover, we collected proteins from cells cultured in six-well plates at confluency. Western blots showed obesity-associated increases in protein expression of p300, CBP and H3K27ac (Fig. 3B), similar to the data we showed above for fetuses from HFD mice. We also found increased expression of PPARγ in UC MSCs from obese mothers compared to those from lean mothers (Fig. 3C). The biological importance of senescent or pre-mature senescent non-cancer somatic cells is not fully understood, but it is reasonable to consider that increased senescence signaling in osteo-progenitors may persist to impair postnatal bone development. To determine whether maternal obesity enhances binding of H3K27ac to PPARγ and cellular senescence-associated target genes (p53 and p21) in human UC MSCs, H3K27ac chromatin immunoprecipitation (ChIP) assays were carried out (Fig. 4A). We used antibody against H3K27ac (recognize both human and mouse) and subsequent PCR amplification. We found that there were pronounced increases in the bindings of H3K27ac to the PPARγ gene and to p53 and p21 in UC MSCs from obese mothers compared to those cells from control lean mothers (Fig. 4B).

### Adult offspring from HFD-induced maternal obese dams have decreased bone formation

Offspring from HFD obese and control diet dams were weaned, and a HFD challenge or control feeding began at 6 weeks of age. Therefore, we have total four groups for male (and female) offspring: control-control (control diet dam and control diet offspring); control-HFD (control diet dam and HFD offspring); HFD-control (HFD dam and control diet offspring) and HFD-HFD (HFD dams and HFD offspring). After 14 weeks of these post weaning diets, the HFD-HFD groups (male and female) gained significantly more weight than any other group (Supplementary Fig. 1).

Adult offspring left tibias were μCT scanned *ex vivo* for determining trabecular and cortical structural changes and BV. In males, compared to control-control as the standard group, the control-HFD group had significantly decreased bone volume (BV/TV), trabecular thickness (Tb.Th) and cortical thickness (Cort.Th); their increased structure model index (SMI) indicated mechanically inferior bone (Table 1). Strikingly, we found even more μCT parameters changed in the HFD-control group in both trabecular and cortical sites in male mice compared with control-control group animals (Table 1). Compared to control-control group, decreased bone volume (BV/TV), connective density (Conne-Dens), trabecular number (Tb.N) and trabecular thickness (Tb.Th), but increased trabecular spaces (Tb.Sp) were found in trabecular site of HFD-control group males (Table 1). At the cortical site, we found increased total CSA, periosteal perimeter, medullary area and endosteal perimeter in the HFD-control male group compared to the control-control group, indicating HFD-induced maternal obesity changes bone structure in offspring. More changes at the trabecular site were found in the male HFD-HFD group compared to the control-control group (Table 1). Overall, the impact of maternal or postnatal HFD was much more profound and numerous in male offspring compared to female offspring (Table 1).

**Table 1:** Micro-CT parameters on trabecula and cortical of tibias of adult offspring from either HFD-induced obese or lean dams.

	Male				Female			
	Control-control	Control-HFD	HFD-control	HFD-HFD	Control-control	Control-HFD	HFD-control	HFD-HFD
Trabecular								
BV/TV	0.28 ± 0.02^c^	0.23 ± 0.04^a^	0.24 ± 0.02^b^	0.22 ± 0.03^a^	0.16 ± 0.06	0.15 ± 0.02	0.14 ± 0.01	0.15 ± 0.02
Conn-dens.	207.3 ± 47.8^c,b^	163.6 ± 33.5^c,b^	153.6 ± 13.2^b^	138.5 ± 6.9^a^	99.3 ± 31.6^c^	57.9 ± 14.1^a^	60.8 ± 13.4^a,b^	68.1 ± 16.7^b^
SMI	1.38 ± 0.29^a^	1.80 ± 0.19^b^	1.55 ± 0.20^a,b^	1.91 ± 0.20^b,c^	2.0 ± 0.56	2.16 ± 0.21	2.31 ± 0.28	2.1 ± 0.2
Tb.N	5.92 ± 0.51^b,c^	5.56 ± 0.39^b^	5.41 ± 0.12^a,b^	5.31 ± 0.10^a^	3.79 ± 0.43	3.47 ± 0.10	3.5 ± 0.15	3.69 ± 0.35
Tb.Th	0.060 ± 0.003^b,c^	0.056 ± 0.003^a,b^	0.055 ± 0.002^a^	0.057 ± 0.004^b^	0.058 ± 0.006^a^	0.065 ± 0.002^b,c^	0.063 ± 0.002^b^	0.062 ± 0.002^a,b^
Tb.Sp	0.152 ± 0.016^a^	0.163 ± 0.016^a,b^	0.167 ± 0.006^b^	0.172 ± 0.006^c^	0.26 ± 0.03	0.28 ± 0.007	0.28 ± 0.02	0.27 ± 0.03
vBMD	891.2 ± 31.4	871.1 ± 3.5	885.2 ± 12.7	886.2 ± 13.1	907.1 ± 22.6	942.3 ± 34.6	922.3 ± 22.2	911.5 ± 15.3
Cortical								
Cort CSA	0.40 ± 0.03	0.38 ± 0.01	0.43 ± 0.04	0.42 ± 0.04	0.34 ± 0.03	0.35 ± 0.02	0.35 ± 0.01	0.33 ± 0.008
Cort.Th	0.21 ± 0.01^b^	0.20 ± 0.006^a^	0.21 ± 0.01^b^	0.21 ± 0.01^b^	0.20 ± 0.01	0.21 ± 0.01	0.21 ± 0.007	0.20 ± 0.004
Total CSA	0.73 ± 0.03^a^	0.71 ± 0.02^a^	0.80 ± 0.05^b^	0.77 ± 0.09^a^	0.62 ± 0.07	0.63 ± 0.04	0.60 ± 0.03	0.60 ± 0.02
Periosteal perim.	2.34 ± 0.14^a^	2.38 ± 0.07^a^	2.53 ± 0.16^b^	2.51 ± 0.32^a,b^	2.13 ± 0.12	2.18 ± 0.07	2.11 ± 0.08	2.08 ± 0.07
Midshaft diam.	0.61 ± 0.03	0.59 ± 0.008	0.62 ± 0.01	0.60 ± 0.02	0.57 ± 0.03	0.57 ± 0.02	0.56 ± 0.008	0.56 ± 0.004
Medullary area	0.29 ± 0.01^a^	0.28 ± 0.01^a^	0.32 ± 0.02^b^	0.31 ± 0.04^a,b^	0.24 ± 0.04	0.24 ± 0.03	0.22 ± 0.02	0.22 ± 0.01
Endosteal perim.	1.44 ± 0.05^a^	1.49 ± 0.06^a^	1.58 ± 0.1^b^	1.54 ± 0.2^a,b^	1.30 ± 0.14	1.32 ± 0.08	1.22 ± 0.08	1.27 ± 0.04
Medullary diam.	0.39 ± 0.02	0.37 ± 0.006	0.40 ± 0.02	0.39 ± 0.007	0.36 ± 0.02^b^	0.35 ± 0.02^b^	0.35 ± 0.02^b^	0.34 ± 0.009^a^

Numbers are mean ± s.d., means with different letters differ significantly (*P* < 0.05, *a* < *b* < *c*) as determined by two-way ANOVA followed by Student-Newman-Keuls post hoc analysis for multiple pairwise comparisons. *n* = 7 per group in males and *n* = 5 per group in females.

BV/TV (%), bone volume/total volume; Conn-Dens (mm), connective tissue density; Cort CSA, cortical cross section area; Cort.Th, cortical thickness; SMI, structure model index; Tb.N (1/mm), trabecular number; Tb.Sp (mm); trabecular separation; Tb.Th (mm), trabecular thickness; vBMD, volumetric bone mineral density.

We performed ELISA measurements of glucose, insulin and bone formation markers (total osteocalcin, carboxylated osteocalcin and bone-specific ALP) in bone marrow plasma collected at the end of the post weaning feeding intervention. We found increased glucose and insulin levels and decreased levels of total osteocalcin and bone-specific ALP in the male HFD-HFD group compared to the control-control group (Table 2). We found decreased total osteocalcin level in the male HFD-control group compared to the control-control group. There was higher insulin level but decreased bone-specific ALP level in the male control-HFD group compared to the control-control group (Table 2). Compared to the significant changes of insulin and bone formation marker levels in male mice, there are differences in levels of those markers from the control-HFD, HFD-control groups compared to those in the control-control female mice, but not statistically different (Table 2). We only found significant changes of insulin and total osteocalcin levels in the female HFD-HFD group compared to the bone marrow plasma levels in the female control-control group (Table 2).

**Table 2:** Bone marrow plasma concentrations of insulin, glucose and bone formation markers.

	Male				Female			
	Control-control	Control-HFD	HFD-control	HFD-HFD	Control-control	Control-HFD	HFD-control	HFD-HFD
Glucose (mg/mL)	0.61 ± 0.03^a,b^	0.59 ± 0.025^a^	0.63 ± 0.01^b^	0.65 ± 0.015^b,c^	0.63 ± 0.015	0.63 ± 0.015	0.62 ± 0.04	0.62 ± 0.025
Insulin (µg/L)	0.19 ± 0.0045^a^	0.21 ± 0.015^b^	0.18 ± 0.015^a^	0.22 ± 0.014^b^	0.19 ± 0.021^a^	0.21 ± 0.009^b^	0.24 ± 0.041^b,c^	0.22 ± 0.002^b^
Total osteocalcin (ng/mL)	38.4 ± 7.58^c^	29.3 ± 8.13^b^	18.7 ± 2.58^a^	17.5 ± 5.23^a^	29.4 ± 5.96^b^	29.2 ± 7.65^b^	24.4 ± 13.1^a^	23.9 ± 4.79^a^
Glu-osteocalcin (ng/mL)	0.53 ± 0.15^c^	0.45 ± 0.10^b^	0.34 ± 0.14^a^	0.42 ± 0.14^b^	0.76 ± 0.21^c^	0.61 ± 0.24^b^	0.44 ± 0.15^a^	0.53 ± 0.14^b^
ALP (µUnits/min)	0.45 ± 0.07^b^	0.31 ± 0.07^a^	0.45 ± 0.08^b^	0.34 ± 0.05^a^	0.41 ± 0.05	0.35 ± 0.08	0.34 ± 0.09	0.43 ± 0.03

Numbers are mean ± s.d., means with different letters differ significantly (*P* < 0.05, *a* < *b* < *c*) as determined by two-way ANOVA followed by Student-Newman-Keuls post hoc analysis for multiple pairwise comparisons. *n* = 7 per group in males and *n* = 5 per group in females.

ALP, alkaline phosphatase; Glu-osteocalcin, carboxylated osteocalcin.

### Increased cell senescence signaling in embryonic osteo-progenitors from HFD dams persisted in bone of adult offspring

**Figure 5:**
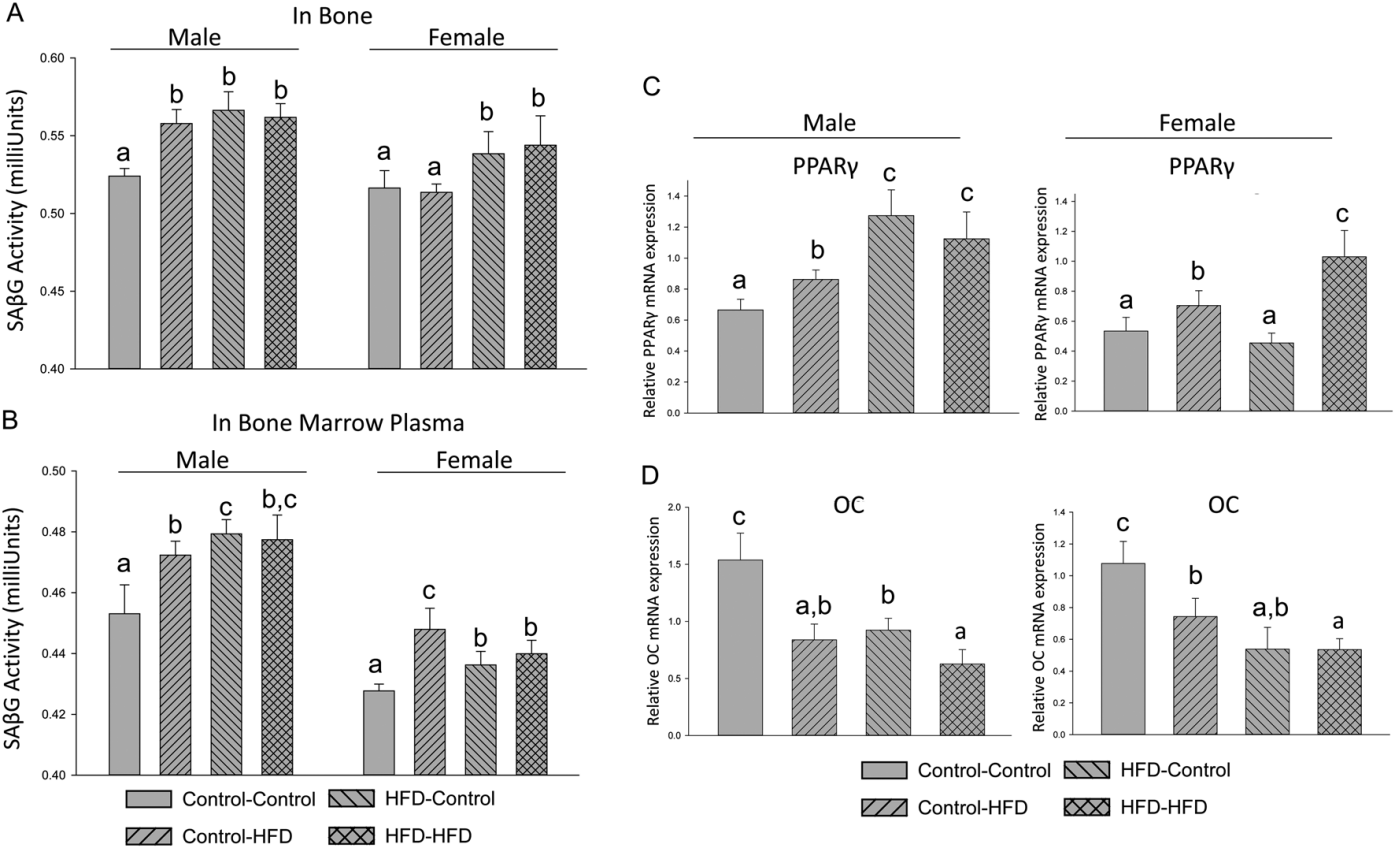
Increased senescence signaling in adult bone of offspring with pre- or postnatal HFD. (A) Total proteins were isolated from femur of all offspring male and female mice after aspiration of bone marrow; SAβG activity measurement in total proteins from all adult offspring groups. (B) SAβG activity measurement in total proteins from bone marrow plasma from all adult offspring groups. (C) mRNA expression of PPARγ and (D) mRNA expression of osteocalcin (OC) in bone from all adult offspring mice. Bars represent means ± s.d.; for *n* = 5–8 animals/group. Means with different letters differ significantly (*P* < 0.05, *a* < *b* < *c*) as determined by two-way ANOVA followed by Student–Newman–Keuls post hoc analysis for multiple pairwise comparisons.

The results of these studies raise two additional questions at the molecular level. First, does the increased senescence signaling in osteo-progenitors from embryos derived from HFD dams persist into bone of adult offspring, i.e., through epigenetic regulation? Second, does increased senescence signaling in osteoblastic cells truly contribute to early bone degeneration or skeletal involution as mentioned previously? Obviously, it will be difficult and complicated to test second question. However, here we have begun to examine the first question. We isolated protein and RNA from mouse offspring bone. To assess if previously observed senescence signaling in fetal cells from HFD obese dams is also occurring in adult offspring bone, we measured SAβG activity in bone and bone marrow plasma. SAβG activity in bone was significantly higher in the male control-HFD, HFD-control and HFD-HFD groups compared to its activity in bone in the control-control group (Fig. 5A). Similar results were found in bone marrow plasma (Fig. 5B).

**Figure 6:**
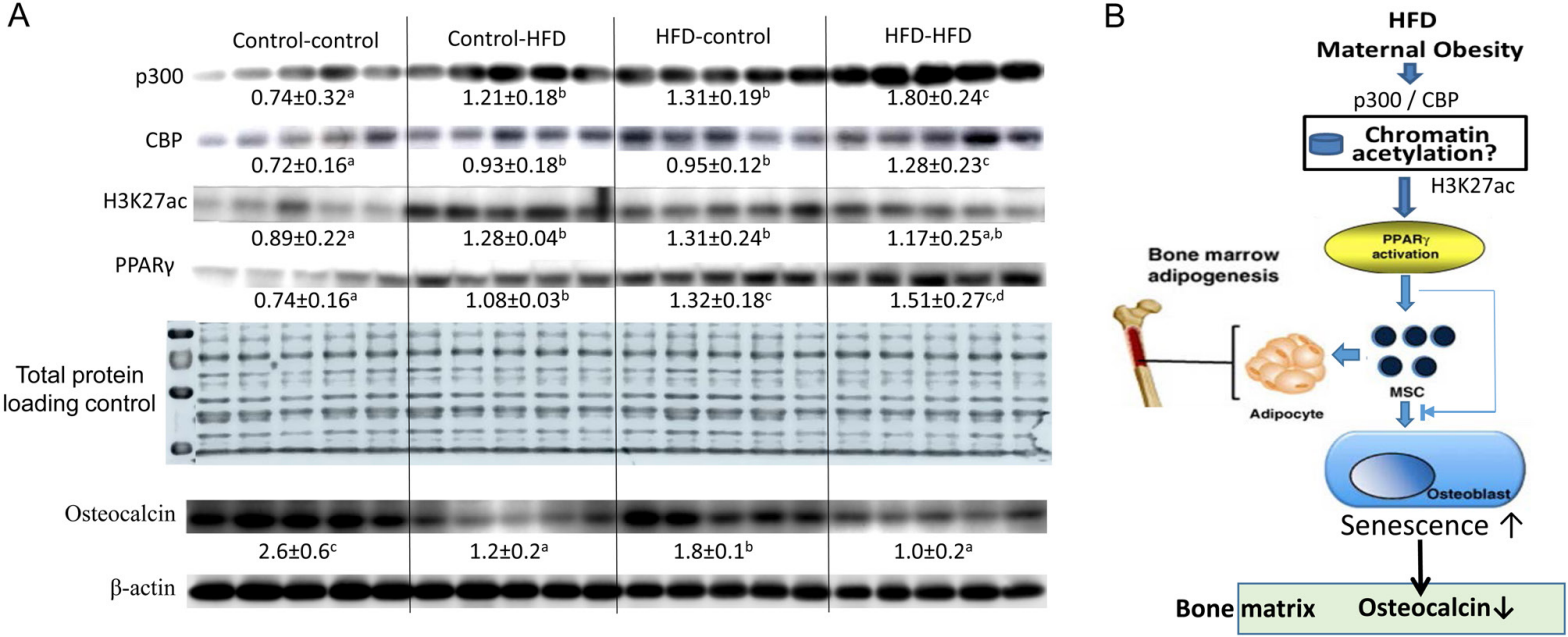
Activated p300 and CBP in adult offspring bone with pre- or postnatal HFD. (A) Total proteins were isolated from femur of all offspring male mice after aspiration of bone marrow; Western blots for p300, CBP, H3K27ac, PPARγ and osteocalcin were performed in total proteins from male adult offspring groups. Numbers under each group blot are mean ± s.d. of band intensities after normalized by control loading proteins (*n* = 5 per group). Means with different letters differ significantly (*P* < 0.05, *a* < *b* < *c* < *d*) as determined by two-way ANOVA followed by Student-Newman-Keuls post hoc analysis for multiple pairwise comparisons. (B) Summary figure and working hypothesis for maternal obesity activating p300/CBP to increase H3K27 acetylation therefore increased PPARγ activation leading to increased senescence signaling and differentiation of more adipocytes from mesenchymal stem cells (MSCs).

As an indirect measure of potential epigenetic events, mRNA expression in bone was evaluated. Transcript abundance of PPARγ in bone in the male control-HFD, HFD-control and HFD-HFD groups was significantly higher compared to its expression in bone from the control-control mice (Fig. 5C), while increased PPARγ mRNA expression was only found in the HFD-HFD group in female adult offspring (Fig. 5C). It was surprising, however, that we found osteocalcin, the mature osteoblast marker, mRNA expression was significantly lower in the control-HFD, HFD-control and HFD-HFD male and female groups compared to their respective control-control groups (Fig. 5D). Gene acetylation and cellular senescence signaling pathway involved in p300, CBP and PPARγ protein expression were also examined in bone from adult male mice. As shown in Fig. 6, p300, CBP, H3K27ac and PPARγ protein expression were clearly increased in bone from the control-HFD, HFD-control and HFD-HFD adult male offspring compared to their expression in bone from the control-control adult offspring (Fig. 6). Consistent with osteocalcin mRNA expression levels, osteocalcin protein expression was significantly lower in the control-HFD, HFD-control and HFD-HFD male groups compared to their respective control-control group (Fig. 6). These data indicated that increased senescence signaling in bone of adult offspring from HFD maternal obese mice, and offspring early life HFD are associated with protein acetylation, i.e., epigenetic regulation.

## Discussion

The maternal environment, in particular, the nutritional status and obesity during pregnancy, has been shown to be important in the developmental trajectory of the fetus and to influence risk for chronic diseases such as diabetes, cardiovascular disease, osteoporosis and cancer in the offspring (Cooper *et al*. 2009, Godfrey *et al*. 2011). Maternal obesity, best known for predisposing offspring to obesity and metabolic diseases, has recently seen increased investigation in both animal models and humans on its effects on offspring tissue phenotype. The human skeleton is known as the largest endocrine organ; however, the fetal skeletal development and adult offspring bone formation associated with maternal obesity are still understudied. We previously hypothesized and provided new evidence (Chen *et al*. 2012, 2016) that the fetal skeleton represents another target for developmental programming and might lead to changes in ability to attain peak bone mass and thus alter the risk of osteoporosis in later life (Bedford *et al*. 2011). In the current study, we have, for the first time, using a mouse model combined with analysis of osteo-progenitors from human obese mothers, provided further evidence that maternal obesity regulates fetal osteoblastic cell senescence signaling and fetal and adult offspring skeletal development. We showed that regardless of postnatal HFD challenge, adult offspring from HFD obese dams have significantly suppressed bone formation. This phenomenon appeared to be much more robust in males compared to females. Such suppressed bone formation in adult offspring from HFD obese dams is a phenomenon of early bone involution/degeneration, and may be in part due to histone acetylation, i.e., epigenetic regulation of genes involved in cell senescence signaling in pre-osteoblasts. Although we have attempted to determine the links between epigenetic regulation of fetal pre-osteoblastic cell senescence signaling and altered embryonic and adult offspring skeletal development, under the condition of maternal obesity, such links may be universal for other tissue development in adult offspring in rodents and perhaps in humans.

The results revealed increased H3K27 acetylation in embryonic osteo-progenitors from HFD-induced obese mouse dams and in osteo-progenitors derived from umbilicus MSCs following delivery by pregnant obese mothers. Increased H3K27 acetylation is associated with increased p300/CBP activity. The mammalian p300/CBP is a pair of ubiquitously expressed, paralogous proteins that belong to a distinct family of histone acetyltransferases (HATs) (Bedford & Brindle 2012). Although increased p300/CBP activity enhances H3K27 acetylation and is consistent with previous evidence (Liu *et al*. 2014), activation of p300/CBP and increased H3K27 acetylation in embryonic osteo-progenitors associated with maternal obesity are new findings. p300/CBP are essential for animal development as deletion of either one in mice leads to early embryonic lethality (Kraus & Wong 2002). These two HATs have been shown to function as transcription co-activators for hundreds of transcription factors including nuclear receptors (NRs) (Chen *et al*. 2015), and here, we have shown PPARγ, peroxisome proliferator-activated receptor gamma, is a nuclear receptor. The role of PPARγ involving adipogenesis is well known; however, more functions of PPARγ on cellular signal transduction have been discovered recently. For example, it has been shown that overexpression or activation of PPARγ will in turn accelerate senescence pathways by inducing p16INK4α expression in a ligand-dependent manner (Tchkonia *et al*. 2010). In this regard, PPARγ was suggested as a molecule linking external or systemic factors (such as diet or obesity) and specific intra-cellular factors (such as the p16 gene) to control cellular senescence.

We have reported previously the effect of maternal obesity on fetal osteoblastic cell senescence pathways in a rat model (Chen *et al*. 2016), and the current results are consistent with those observations in general terms. Obesity may be considered to be associated with an increase in cellular senescence through systemic low-grade chronic inflammation (Kereliuk *et al*. 2017). From data presented in our current report, it supports that attenuation of physiological resolution of inflammation or low-grade inflammation occurred in fetal skeletal tissues from HFD-induced obese dams. Our results in mouse embryonic osteo-progenitors are consistent with previous evidence that cellular senescence promotes chronic inflammation through the SASP (Coppé *et al*. 2008). Moreover, we believe that increased cell senescence signaling in embryonic mouse calvarial cells and human MSCs and offspring in bone associated with maternal obesity are atypical. Specifically, in maternal obesity-associated embryonic osteogenic cells, increased PPARγ may interact with p53 and p21 to control senescence signaling in these cells. Supporting such a scenario, we have shown that HFD-induced postnatal obesity increases PPARγ expression to promote p53/p21-mediated cell senescence signaling in bone and osteoblastic cells (Chen *et al*. 2015). p300/CBP and increased H3K27 acetylation may be upstream of the team of PPARγ/p53/p21 in the involvement of embryonic osteogenic cell senescence signaling. Although our data suggest a novel phenomenon of maternal programming of pre-osteoblastic cell senescence signaling, obviously, signaling molecules we presented here are not bone cell type specific. Those cellular senescence-associated signaling molecules are expressed in variety of tissues; therefore, our data might implicate a universal and potential mechanistic explanation for poorly defined maternal pathophysiologic programming in other tissue systems.

While human studies, especially in postnatal offspring bone development, are important to the field, it is difficult to control for confounding factors over a lengthy and expensive study period, and genetic variability adds a high level of complexity. Therefore, rodent models with a common genetic background and carefully controlled dietary and activity conditions are useful for examining how altered HFD or obesity during gestation or early postnatal life influences the development of bone associated with obesity in the offspring. On the other hand, there are also significant limitations that need to be considered when using animals to model human pregnancy that can impact the generalizability of the findings. For example, the number of offspring per pregnancy, placentation, gestational length, parturition and different windows of fetal and neonatal cellular differentiation and organogenesis (Kereliuk *et al*. 2017). In addition, the normal macronutrient intake for rodents as a percent of total energy intake can be quite different from humans. Nonetheless, recent results from the Danish National Birth Cohort show that maternal poor-quality diet (i.e., a dietary pattern in which the most abundant food items are potatoes, french fries, white bread, pork, beef veal, mixed meat, cold meat and dressing sauce) is associated with significantly increased risk of childhood fractures in offspring (Petersen *et al*. 2015), supporting the idea that offspring skeletal health is influenced by the gestational environment and maternal diet (Bedford *et al*. 2011). Although human UC MSCs as we used in our study may not exactly represent fetal bone phenotype and bone progenitor functions, these cells are the most accessible fetal mesenchymal osteo-progenitor cells in infants during delivery. Accelerated senescence signaling pathway in those cells from obese mothers was very close finding to embryonic calvarial cells from HFD-induced obese mouse dams. It is acknowledged that despite clear outcomes, our findings in human cells are limited to a small sample size. Therefore, future clinical investigations of bone phenotype of offspring from obese mothers compared to those from lean mothers will be of interest.

Although mechanistic processes of normal development of bone and adipose tissue during early life are not well characterized, our current study elucidated the effect of maternal HFD on postnatal long bone development in mice. It is known, however, that maternal obesity enhances MSCs to form more committed pre-adipocytes and white adipocytes, which lead to adiposity in specific depots in offspring. This finding is consistent with our data in MSCs in our previous report from pregnant obese women and in a rat model (Chen *et al*. 2016). Previous evidence presented by us also suggested an epigenetic basis of such unbalanced differentiation potential between adipogenesis and osteoblastogenesis (Chen *et al*. 2016). This observation seems to be true from our current study; our data indicated that HFD in offspring from prenatal HFD dams significantly gain more weight than those from prenatal control diet dams. Moreover, significant changes in bone marrow plasma glucose and insulin levels and decreased bone formation marker osteocalcin and bone-specific ALP suggest HFD maternal obesity-related shifts in anabolic and catabolic responses that control bone homeostasis and explain postnatal bone phenotype characterized by µCT. However, it was surprising, without postnatal HFD challenge, weight gains in offspring from HFD obese mice had similar trends compared to those offspring from control diet dams (within postnatal study duration 20 weeks), yet, bone loss occurred. Such bone loss in offspring from HFD maternal obese mice is a phenomenon of early bone degeneration or early involution of bone formation. Indeed, HFD+control group had more effects on trabecular number than control+HFD group, while HFD+HFD had the greatest effect. We provided further data suggesting an association with accelerated bone senescence and epigenetic regulation. More *in vivo* studies may be needed to investigate that altered and epigenetically regulated senescence signaling in their precursors may interfere with adipogenic and osteogenic signals and therefore determine cell fate to either differentiate toward adipocytes or osteoblasts (Zhang *et al*. 2011, 2013). Moreover, our data showed that there may be difference in bone phenotype in offspring between male and female in response to either pre or postnatal HFD. Such gender differences in response to HFD have been shown in brown and white adipose tissue development (Strakovsky *et al*. 2014); sex chromosome or postnatal physiologic estrogen signaling may influence or dilute HFD-induced epigenetic regulation on tissue development. Nonetheless, it will be interesting for our future studies to determine in more detail of how sex chromosome or postnatal physiologic estrogen signaling has an effect on HFD-induced decreased postnatal skeletal development.

In summary, we have presented evidence suggesting epigenetic regulation of HFD-induced maternal obesity on both fetal and adult offspring skeletal development. We found CBP/p300 activation leads to H3K27 acetylation, which may increase cell senescence-related gene and PPARγ expression in EOCCs from HFD-obese dams, and in human UC MSCs isolated following delivery by obese and lean mothers. Adult offspring from HFD obese dams with standard control diet showed significantly suppressed bone formation. Such early involution of bone formation of adult offspring from HFD-obese dams is likely in part due to histone acetylation, i.e., epigenetic regulation of genes involved in cell senescence signaling in pre-osteoblasts from prenatal period.

## Supplementary Material

Supporting Figure 1

Supporting Table 1

## Declaration of interest

The authors declare that there is no conflict of interest that could be perceived as prejudicing the impartiality of the research reported.

## Funding

This work was supported by the United States Department of Agriculture (USDA)/Agricultural Research Service (ARS), project number # USDA-ARS Project 6026-51000-010-05S, to the Arkansas Children’s Nutrition Center.

## Author contribution statement

J R C contributed to experiment design, performing experiments (senescence signaling determination, etc.), data analysis and writing paper. O P L contributed by performing experiments (Western blots, real-time PCR, ChIP, etc.) and data analysis. H J Z contributed by performing experiments (Western blots and real-time PCR, etc.). A W A contributed by performing experiments (micro-CT). K S contributed to experimental design.
