# Supplementary material for: Maternal obesity impairs skeletal development in adult offspring
Source: J Endocrinol. 2018 Jul 24;239(1):33–47. doi: 10.1530/JOE-18-0244 (PMC6145139; doi:10.1530/JOE-18-0244)
Supplement: Supporting Figure 1 [file joe-239-33-s001.pdf]

Supplemental Figure 1: experimental design

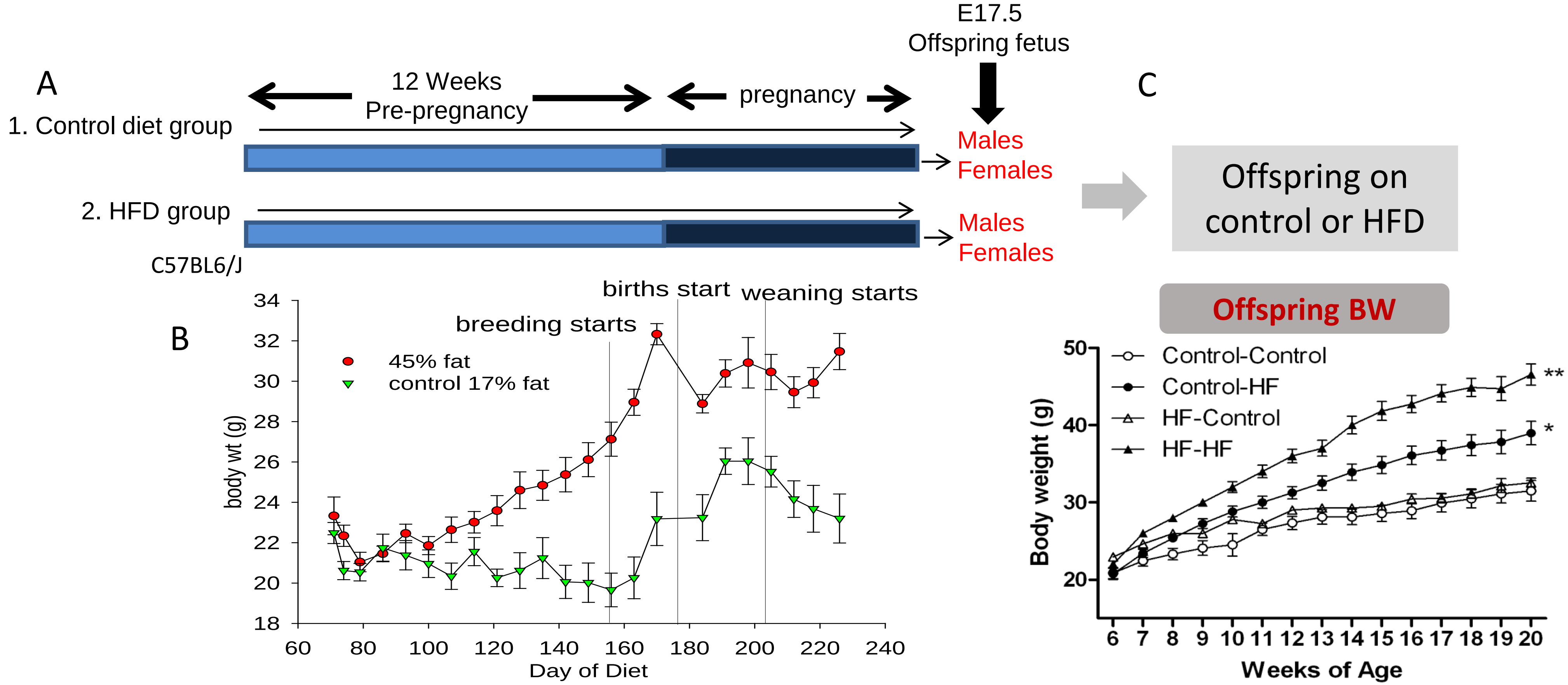


*Supplemental Figure 1, A, 6 wks old C57BL6/J female mice were either fed high fat diet (HFD): 220 kcal/kg$^{3/4}$·day, 20% protein (casein), 35% carbohydrate (dextrose and maltodextrin), and 45% fat (corn oil) or control lab rodent diet for 12 wks (n=10). After 12 wks of HF or control diets, these mice were met with control diet male mice, HF or control diet were continued during pregnancy. At embryonic age 17.5 (E17.5), half dams were killed and fetuses were taken for analysis, another half of dams were kept for offspring study. B, dams body weight. C, offspring body weight tracking with or without HFD challenge for 14 wks.*
