# Supplementary material for: Maternal obesity impairs skeletal development in adult offspring
Source: J Endocrinol. 2018 Jul 24;239(1):33–47. doi: 10.1530/JOE-18-0244 (PMC6145139; doi:10.1530/JOE-18-0244)
Supplement: Supporting Table 1 [file joe-239-33-t001.pdf]

Supplemental Table 1: Primer information for ChIP assay

Gene	Forward primer 5'-3'	Reverse primer 5'-3'	Position
**p53**	GTGATAAGGGTTGTGAAGGAG	GGGTGTGGATATTACGGAAAGCCT	-691 -623
**p21**	GGAGGCAAAAGTCCTGTGTTC	GGAAGGAGGGAATTGGAGAG	-306 -108
**PPARG**	ACATCTTGGGAAGACGGCCTG	CAGGCTACCTGGTGTCGTTTGC	-368 -166

Human ChIP primers

Gene	Forward primer 5'-3'	Reverse primer 5'-3'	Position
**p53**	GCTGTGCAATTAAAGGCTGTGA	TGTTCTCCGAGATACTTGGTATCG	-789 -686
**p21**	ACAGAAACCCTAAATGTGGCATTC	GGTCAGTGAGCTCCAGGCTCTA	-987 -887
**PPARG**	CCAAATACGTTTATCTGGTGTTTC	CGTTGCTACATTGTCTCGC	-437 -257

Mouse ChIP primers
